# Measuring school level attributable risk to support school-based HPV vaccination programs

**DOI:** 10.1186/s12889-022-13088-x

**Published:** 2022-04-25

**Authors:** C. Vujovich-Dunn, H. Wand, J. M. L. Brotherton, H. Gidding, J. Sisnowski, R. Lorch, M. Veitch, V. Sheppeard, P. Effler, S. R. Skinner, A. Venn, C. Davies, J. Hocking, L. Whop, J. Leask, K. Canfell, L. Sanci, M. Smith, M. Kang, M. Temple-Smith, M. Kidd, S. Burns, L. Selvey, D. Meijer, S. Ennis, C. Thomson, N. Lane, J. Kaldor, R. Guy

**Affiliations:** 1 University of New South Wales, Kirby Institute, Kensington, Australia; 2 Australian Centre for the Prevention of Cervical Cancer, Population Health, East Melbourne, Victoria, Australia; 3 University of Melbourne, Melbourne School of Population and Global Health, Carlton, VIC Australia; 4 University of Sydney, Northern Clinical School, Sydney, Australia; 5 Women and Babies Research, Kolling Institute, Northern Sydney Local Health District, Sydney, Australia; 6 School of Population Health, University of New South Wales, Kensington, Australia; 7 National Centre for Immunisation Research and Surveillance, Sydney, Australia; 8 Australian National University, National Centre for Epidemiology & Population Health, Canberra, Australia; 9 Department of Health and Human Services, Tasmanian Government, Hobart, Australia; 10 Communicable Diseases Branch, NSW Health, St Leonards, New South Wales, Australia; 11 University of Sydney, Sydney School of Public Health, Camperdown, NSW Australia; 12 Communicable Disease Control Directorate, Department of Health, Western Australia, East Perth, Australia; 13 University of Sydney, Specialty of Child and Adolescent Health, Faculty of Medicine and Health, Sydney, Australia; 14 Children’s Hospital Westmead, Sydney Children’s Hospitals Network, Westmead, Australia; 15 Menzies Institute for Medical Research, University of Tasmania, Tasmanian, Australia; 16 Menzies School of Health Research, Charles Darwin University, Cairns, QLD Australia; 17 University of Sydney, Sydney Nursing School, Faculty of Medicine and Health, Camperdown, NSW Australia; 18 The Daffodil Centre, University of Sydney, A Joint Venture With Cancer Council NSW, Sydney, Australia; 19 University of Melbourne, Medicine, Dentistry and Health Sciences, Carlton, VIC Australia; 20 School of Public Health, University of Sydney, Sydney, New South Wales Australia; 21 University of Sydney, Westmead Clinical School, Sydney, New South Wales Australia; 22 Flinders University, Southgate Institute for Health, Society and Equity, Bedford Park, South Australia Australia; 23 Curtin University, School of Population Health, Bentley, WA Australia; 24 University of Queensland, School of Public Health, St Lucia, QLD Australia; 25 Immunisation Unit, Health Protection NSW, St Leonard’s, New South Wales, Australia

**Keywords:** HPV vaccines, Primary prevention, Cervical cancer, Immunisation programs, School-based, Health equity

## Abstract

**Background:**

In Australia in 2017, 89% of 15-year-old females and 86% of 15-year-old males had received at least one dose of the HPV vaccine. However, considerable variation in HPV vaccination initiation (dose one) across schools remains. It is important to understand the school-level characteristics most strongly associated with low initiation and their contribution to the overall between-school variation.

**Methods:**

A population-based ecological analysis was conducted using school-level data for 2016 on all adolescent students eligible for HPV vaccination in three Australian jurisdictions. We conducted logistic regression to determine school-level factors associated with lower HPV vaccination initiation (< 75% dose 1 uptake) and estimated the population attributable risk (PAR) and the proportion of schools with the factor (school-level prevalence).

**Results:**

The factors most strongly associated with lower initiation, and their prevalence were; small schools (OR = 9.3, 95%CI = 6.1–14.1; 33% of schools), special education schools (OR = 5.6,95%CI = 3.7–8.5; 8% of schools), higher Indigenous enrolments (OR = 2.7,95% CI:1.9–3.7; 31% of schools), lower attendance rates (OR = 2.6,95%CI = 1.7–3.7; 35% of schools), remote location (OR = 2.6,95%CI = 1.6–4.3; 6% of schools,) and lower socioeconomic area (OR = 1.8,95% CI = 1.3–2.5; 33% of schools). The highest PARs were small schools (PAR = 79%, 95%CI:76–82), higher Indigenous enrolments (PAR = 38%, 95%CI: 31–44) and lower attendance rate (PAR = 37%, 95%CI: 29–46).

**Conclusion:**

This analysis suggests that initiatives to support schools that are smaller, with a higher proportion of Indigenous adolescents and lower attendance rates may contribute most to reducing the variation of HPV vaccination uptake observed at a school-level in these jurisdictions. Estimating population-level coverage at the school-level is useful to guide policy and prioritise resourcing to support school-based vaccination programs.

**Supplementary Information:**

The online version contains supplementary material available at 10.1186/s12889-022-13088-x.

## Introduction

Human papillomavirus (HPV) infection causes nearly all cervical cancers, with cervical cancer the fourth most common cause of cancer incidence and mortality in women worldwide [1]. HPV is also responsible for 90% of anal, 60% of penile and 30% of oropharyngeal cancers globally [2, 3]. Since the 1970s the incidence of oropharyngeal cancer has substantially increased among younger age groups in the United States, Canada, parts of Europe and Australia [2, 4, 5]. In addition, HPV causes 90% of genital warts, which prior to vaccine introduction was the most commonly managed sexual health condition in Australia [2]. HPV-related cancers and genital warts have significant adverse impacts on reproductive health and quality of life and are costly to manage [6, 7].

The first HPV vaccine providing protection against oncogenic types of HPV and, in turn, invasive cervical cancer, other cancers and genital warts was registered in Australia in 2006 [8, 9]. Numerous studies since have demonstrated the population effectiveness of the vaccine in reducing genital warts [10, 11], high-grade precancerous cervical lesions [12–14] and, more recently, invasive cervical cancer [15]. As of late 2019, over 124 countries had implemented a national HPV vaccination program [16]. HPV vaccination programs are targeted at young adolescents, as the vaccine is most effective prior to initiation of sexual activity and exposure to the virus [17, 18]. In November 2020, the World Health Organisation (WHO) launched the ‘Global Strategy to accelerate the elimination of cervical cancer as a public health problem’, which is underpinned by the three pillars of HPV vaccination, cervical screening using HPV testing (or equivalent), and treatment [19]. The strategy calls for the achievement of HPV vaccination coverage of 90% of girls by age 15 years (based on the coverage necessary in countries with a single-sex vaccination program) [19]. This goal was set based on the effectiveness of HPV vaccines [13, 14, 20], and cervical screening using HPV testing.

Voluntary on-site school-based HPV vaccination programs have been successful in achieving high rates of vaccination coverage in adolescents in Australia, Canada, several European countries and several low- and middle-income countries [21–23]. Australia was the first country to implement a fully funded, national, school-based HPV vaccination program, and since its introduction has achieved high coverage, reaching just over 80% of eligible students in 2017 for the 3-dose course [24], before switching to a 2-dose course in 2018. The school-based HPV vaccination program operates at the beginning of secondary school, either in Year 7 or Year 8 (11 to 14 years of age), for boys and girls. Public Health units or local councils are responsible for administering the vaccines, in partnership with schools. In Australia, adolescents access the HPV vaccine primarily through their schools, however students who miss doses through the school-based program are eligible to receive the vaccine through primary care up until the age of 19 years.

Despite the high HPV vaccination coverage achieved in Australia, programmatic reports have indicated there is variation in coverage between jurisdictions and also between smaller geographical areas within jurisdictions [25, 26]. Recently, we examined variation at the schoollevel, and identified school-level factors associated with lower initiation of HPV vaccination across 3 Australian states [27]. Further investigation of these data at a school level demonstrated 25% of schools had low initiation coverage (< 75% dose 1 uptake) and there were multiple school-level factors associated with lower initiation coverage [27]. Here we extend this analysis to calculate the population-attributable risk (PAR) [28], which accounts for both the strength of the association and the prevalence of the risk factor in the population. While PAR is traditionally used to calculate risk for individuals in a population, our unit of measure is at the school level and, thus, hereafter we will refer to the PAR as school-level attributable risk. Measuring school-level attributable risk may determine where to focus interventions to address variations in HPV vaccination coverage at the school level, and, in turn, ensure all adolescents have equal opportunity to be offered HPV vaccination initiation, irrespective of which school they attend. Coverage is used in this context to describe the percentage of the community who received a vaccine (population level), while uptake is used in the context of a person accepting the vaccine (individual level). Although we are examining school- level factors related to uptake, there are a variety of factors that influence vaccination uptake including sociodemographic characteristics such as income, geographic location, education and occupation; access to health care and other organisational factors; social environmental factors including media influence and social norms; individual child characteristics; individual parental factors including educational level, ethnicity, religious beliefs, and knowledge regarding HPV and cervical cancer; and trust in the pharmaceutical industry and government [23, 29–39]. This method therefore does not assess risk factors at the individual level, but rather provides an indication of school-level factors that can inform interventions and future research.

To our knowledge, the PAR method has not been used previously to understand the contribution of factors associated with vaccine uptake at the school level, with the few studies using this method focused on vaccination uptake in the United States health care setting [40–42], no studies have calculated school-level attributable risk. Here we aim to determine the school-level attributable risk of school-level factors associated with lower HPV vaccination initiation and estimate the school-level attributable risk using the odds ratio and school-level factor prevalence.

## Methods

### Study design and context

We conducted an ecological analysis of HPV vaccine initiation coverage across three Australian states – New South Wales (NSW), Tasmania and Western Australia (WA) – with the school as the unit of analysis.

### Study population

We included all secondary schools, which provide education to adolescents (approximately 12–18 years of age), in the three states for which year-specific student enrolment numbers and number of delivered HPV vaccination doses were available for 2016. We restricted our analysis to schools with vaccine-eligible enrolment numbers of at least 10 students.

### Study outcome

The primary outcome was the proportion of adolescents in each school who initiated the HPV vaccination course (1 or more doses of the quadrivalent HPV vaccine). As the focus of the analysis was school-level coverage (rather than population-level coverage), only doses recorded by the National HPV Vaccination Program Register (NHPVR) as having been delivered in school and which could be attributed to a school and year level were included in the analysis. Initiation coverage for a school was calculated as the number of dose 1 vaccinations reported to the NHPVR divided by the student enrolments for the year level of program delivery. Low initiation coverage was defined as < 75% dose 1 uptake of the first dose.

### Data sources

We obtained data from the following four statutory bodies to calculate the study outcomes and covariates. All datasets were at the school or postcode level of the school in 2016.

**1. National HPV Vaccination Program Register (NHPVR):** the national HPV vaccination registry (closed at the end of 2018), which collected details about HPV vaccinations given in Australia [43]. We obtained vaccination doses given by cohort year and school name.

**2. Jurisdictional health departments:** responsible for immunisation programs, and collection of individual-level data on HPV doses delivered in schools and enrolment data. We obtained school enrolment data for each school grade in which the vaccine program was delivered (Year 7 in two jurisdictions, Year 8 in one jurisdiction). Enrolment data collected from New South Wales (NSW), Western Australia (WA) and Tasmania included graded schools (which denoted school year) for mainstream schools and special education schools. WA also included ungraded ‘special education’ schools (schools that do not place students in a specific year level).

**3. The Australian Curriculum, Assessment and Reporting Authority (ACARA):** an independent statutory authority responsible for the development of a national curriculum, which collects and reports characteristics of schools and their students [44]. We obtained total enrolments for the entire school (school size); student attendance rate (defined as student days attended as a proportion of total possible student days); co-educational status (i.e. whether the school enrols both boys and girls); special education status (i.e. schools catering for students with special educational needs, or mainstream schools); the percentage of students identified as Indigenous students; and the percentage of students with a language background other than English at home.

**4. Australian Bureau of Statistics:** a national statutory body which collects demographic data from Australian residents by a census every 5 years. We obtained data on the Socio-Economic Indexes for Areas Index of Relative Socio-Economic Disadvantage (a standard area-level measure of disadvantage summarising a range of information about economic and social conditions of households within an area) at the postcode level [45], and for remoteness (five classes of remoteness based on a measure of relative access to services: major city, inner regional, outer regional, remote, and very remote) at the postcode level [45].

### Statistical analysis

We merged the datasets above using the school name and postcode. For unmatched schools, we then utilised probabilistic matching using the STATA ‘reclink’ command. Remaining records were reviewed manually, with only plausible matches included.

We conducted univariate logistic regression analysis to identify sociodemographic and school-related factors associated with the primary outcome of low HPV initiation. Sociodemographic variables included the percentage of enrolled students who identified as Indigenous, the percentage of enrolled students with a language background other than English (LBOTE), and the SEIFA IRSD defined by postcode of the school [45]. School-related variables included co-educational status (both boys and girls vs single sex), special education status (catering for special educational needs vs mainstream), geographical location of the school (major city, inner regional, outer regional, remote, or very remote), size of the school (based on total enrolments), attendance (percentage of all possible school days attended), and school affiliation (independent, Catholic, government). We categorised the continuous measurements (school size, Indigenous enrolment, language background other than English, socioeconomic disadvantage score and attendance) using data-driven tertiles. We did not conduct a multivariate analysis due to previously known high levels of collinearity/multicollinearity between the school-level characteristics [27], but instead accounted for the collinearity when calculating the combined school-level attributable risk.

### School-level attributable risk

We estimated school-level impacts of each school-level characteristic on low-coverage schools in the dataset by combining the odds ratios and the prevalence of the school-level characteristics distributed across the schools as described in Table 1. Briefly, we calculated the school-level attributable risk percentage for each characteristic and 95% confidence intervals (CI) individually, as well as their combined impact in a multifactorial setting accounting for their correlation structure [46]. We adjusted for school affiliation, rather than including it as a covariate, to maintain anonymity as requested by stakeholders in the education sector. Analyses were conducted using STATA IC v14.2 (StataCorp, College Station, TX); school-level attributable risk percentages were calculated using SAS version 9 (SAS Inc., Cary, NC). Appendix 1 illustrates the calculation of the school-level attributable risk and the 95% CI for school size as an example variable using the fully documented, publicly available macro (https://www.hsph.harvard.edu/donna-spiegelman/software/par/).

### Ethical approval

Ethical approval was provided by the Human Research Ethics Committees of the University of New South Wales (HC17632), the Australian National University (2017/516), the University of Tasmania (1320/17), the Aboriginal Health and Medical Research Council of New South Wales (1320/17), the Aboriginal Health Council of Western Australia (818), and the Department of Health of Western Australia (RGS0000000456).

## Results

### Characteristics of schools

Of 1325 schools, we excluded 39 schools for which we had enrolment data but no matching school vaccination data, leaving 1286 schools. Of the 1286 schools included, 86% were co-educational, 58% were in major cities, 35% had low attendance rates (< 87% of all possible school days attended), 33% were small (enrolments of 11–383 total students in the school), 33% were located in areas in the most socioeconomically disadvantaged tertile, 31% had a high proportion of Indigenous enrolments, and 8% were special education schools. There were 327 schools considered as low coverage.

### Factors associated with lower initiation

The median school-level HPV vaccination initiation (i.e. dose 1) coverage was 84.7% (IQR 75.0–90.4%), with 327 (25%) schools classified as having low initiation at ≤ 75% coverage. The school-level factors most strongly associated with lower initiation in the univariate analyses and their prevalence were: small size (OR 9.3, 95%CI = 6.1–14.1, 33% of schools, *n* = 419), serving adolescents with special education needs (OR 5.6, 95%CI = 3.7–8.5, 8%, *n* = 8), a higher proportion of Indigenous adolescents (OR 2.7, 95%CI = 1.9–3.7, 31%, *n* = 394), lower attendance rates (OR 2.6, 95%CI = 1.7–3.7, 35%, *n* = 451), location in remote areas (OR 2.6, 95%CI = 1.6–4.3, 6%, *n* = 73) and location in lower socioeconomic areas (OR 1.8, 95% = 1.3–2.5, 33%, *n* = 428) (Table 1). There were strong correlations between these characteristics (Table 2).

**Table 1:** Associations between school characteristics and low in-school HPV vaccine initiation, 2016

**Variables**		**HPV vaccination coverage (dose 1)**		**Univariate analysis**		
	***n* (%) of schools**	**Schools with low initiation (< 75%)**	**% of school low initiation**	**OR**	***p*-value**^**a**^	**95% CI**
**School size**	1256					
** Small (11–383)**	**419 (33%)**	**175**	**41.8%**	**9.3**	** < 0.001**	**6.1, 14.1**
Medium (384–844)	420 (33%)	105	25.0%	4.3	** < 0.001**	**2.8, 6.6**
Large (845–2735)	417 (33%)	30	7.2%	Ref		
**Special education status**	1281					
Mainstream school	1178 (92%)	260	22.1%	Ref		
**Special education school**	**103 (8%)**	**63**	**61.2%**	**5.6**	** < 0.001**	**3.7, 8.5**
**Co-educational status**	1256					
**Co-educational school**	**1105 (88%)**	**293**	**26.5%**	**2.8**	** < 0.001**	**1.7, 4.8**
Single-sex school	151 (12%)	17	11.3%	Ref		
**% Indigenous enrolment**	1189					
Low (0%–2%)	452 (38%)	76	16.8%	Ref		
Medium (3%–8%)^c^	343 (29%)	73	21.3%	1.3	0.110	0.9, 1.9
**High (9%–100%)**	**394 (33%)**	**138**	**35.0%**	**2.7**	** < 0.001**	**1.9, 3.7**
**Remoteness**	1281					
Major cities	751 (59%)	157	20.9%	Ref		
Inner regional	288 (22%)	91	31.6%	1.7	** < 0.001**	**1.3, 2.4**
Outer regional	169 (13%)	45	26.6%	1.4	0.105	0.9, 2.0
**Remote and very remote**	**73 (6%)**	**30**	**41.1%**	**2.6**	** < 0.001**	**1.6, 4.3**
**Attendance rate**	1144					
**Low (29%–87%)**	**451 (39%)**	**143**	**31.7%**	**2.6**	** < 0.001**	**1.7, 3.7**
Medium (88%–91%)	420 (37%)	72	17.1%	1.1	0.542	0.8, 1.7
High (92%–97%)	273 (24%)	42	15.4%	Ref		
**Socioeconomic disadvantage, SEIFA IRSD score**	1280					
**Most disadvantaged (SEIFA 604–967)**	**428 (33%)**	**125**	**29.2%**	**1.8**	** < 0.001**	**1.3, 2.5**
Less disadvantaged (SEIFA 698–1016)	428 (33%)	120	28.0%	1.7	**0.001**	**1.3, 2.4**
Least disadvantaged (SEIFA 1017–1128)	424 (33%)	78	18.4%	Ref		
**Language -Background-Other-Than-English enrolment**	1250					
**Low (0%–6%)**	**455 (36%)**	**132**	**29.0%**	**1.3**	**0.072**	**1.0, 1.8**
Medium (7%–22%)	380 (30%)	77	20.3%	0.8	0.255	0.6, 1.2
High (23%–100%)	415 (33%)	98	23.6%	Ref		

*CI* Confidence interval, *HPV* Human papillomavirus, *IRSD* Index of Relative Socio-Economic Disadvantage, *LBOTE* Language background other than English, *OR* Odds ratio, *SEIFA* Socio-Economic Indexes for Areas

^a^The overall *p*-value is based on the test for heterogeneity

^b^Variables in bold were included in the final reduced model based on their overall significance at the *p* < 0.05 level. The confidence intervals and p-values for variables eliminated from the final model were obtained by adding each variable to the final reduced model

^c^ In Australia 3.3% of the population Identify as Aboriginal

**Table 2:** Correlations between continuous and ordinal categorical covariates (Spearman's rank correlation coefficients), schools in 2016 HPV vaccination program year

	**Indigenous student enrolments (%)**	**Language background other than English student enrolments (%)**	**School postcode SEIFA IRSD score**	**School size**	**Attendance rate (%)**	**Remoteness (1, major cities – 4, remote)**
Indigenous student enrolments (%)						
Language background other than English student enrolments (%)	− 0.4742					
School postcode SEIFA IRSD score	− 0.5447	0.2928				
School size	− 0.5286	0.3557	0.4054			
Attendance rate (%)	− 0.7075	0.2512	0.4518	0.4003		
Remoteness (1, major cities – 4, remote)	0.5991	− 0.5787	− 0.4975	− 0.5529	− 0.3299	

*HPV* Human papillomavirus, *IRSD* Index of Relative Socio-Economic Disadvantage, *SEIFA* Socio-Economic Indexes for Areas

### School-level attributable risk

The school-level attributable risk was based on the 327 low coverage schools. Characteristics which accounted for the largest proportion of low initiation coverage among the low coverage schools were: smaller size (small and medium), with a school-level attributable risk of 79% (95%CI = 76–82), higher Indigenous enrolments (38%, 95%CI = 31–44), lower attendance rates (37%, 95%CI = 29–46), location in postcodes with higher rates of disadvantage (34%, 95%CI = 28–41), serving adolescents with special education needs (27%, 95%CI = 23–31) and location in remote postcodes (24%, 95%CI = 19–29) (Table 3). Including all these school-level factors in the combined model, the school-level attributable risk was 90%, indicating that these factors account for 90% of schools with low HPV vaccination initiation coverage.

**Table 3:** Ranked individual impact of each school characteristic and school-level attributable risk %

School characteristic	School-level attributable risk %
**Overall school size (total school enrolment)**	**79% (76%–82%)**
Small (11–383)	56% (53%–60%)
Medium (384–844)	23% (20%–26%)
Large (845–2735)	Ref
**Overall % Indigenous enrolment**	**38% (31%–44%)**
Low (0%–2%)	Ref
Medium (7%–22%)	6% (4%–8%)
High (9%–100%)	32% (27%–37%)
**Overall attendance rate (% of all possible school days attended)**	**37% (29%–46%)**
Low (29%–87%)	34% (29%–40%)
Medium (88%–91%)	3% (2%–5%)
High (92%–97%)	Ref
**Overall relative socioeconomic disadvantage score (postcode)**	**34% (28%–41%)**
Most disadvantaged (604–967)	18% (15%–22%)
Less disadvantaged (698–1016)	16% (13%–20%)
Least disadvantaged (11,017–1128)	Ref
**Overall special education status**	**27% (23%–31%)**
Mainstream school	Ref
Special education school	27% (23%–31%)
**Overall remoteness**	**24% (19%–29%)**
Major cities	Ref
Inner regional	13% (10%–16%)
Outer regional	4% (3%–5%)
Remote and very remote	7% (6%–9%)

## Discussion

In our multi-state school-level analysis of HPV vaccination initiation coverage, we found the school-level factors most strongly associated with lower dose 1 uptake were: smaller numbers of enrolled students, a student population comprising adolescents with a disability, a higher proportion of Indigenous adolescents, lower attendance rates, a location in remote areas and a location in lower socioeconomic areas. Taking both the odds ratios and the school factor prevalence into account, the school-level attributable risk analysis shows that the characteristics that accounted for the largest proportion of low coverage schools were small size, higher Indigenous enrolments and lower attendance rates.

Based on the highest odds ratio only, small schools were most strongly associated with lower school-level HPV vaccination initiation. As smaller schools (small and medium schools combined) were common, they also had the highest school-level attributable risk at 79%. We are only aware of one other study in the UK which has examined HPV vaccination coverage correlated at a school level, which found that smaller schools achieved lower coverage compared to other schools in the sample [47]. The UK study did not differentiate initiation coverage from completion though, but hypothesised that lower coverage may be due to less resource prioritisation as it may be more efficient to focus on increasing vaccination in larger schools rather than smaller ones [47].

Schools with a higher proportion of Indigenous adolescent enrolment also had a high odds ratio; as such schools were more common, the school-level attributable risk was 38%. Another population-based study in four Australian states and territories demonstrated that, in 2015–2016, first dose coverage exceeded 80% for Indigenous adolescents and was similar to non-Indigenous adolescents; however, this study only included one of the three jurisdictions in our study, highlighting the variation across jurisdictions in Australia [48]. These findings are important as, in Australia, there are important disparities in the burden of disease from cervical cancer, with higher incidence and mortality documented in Indigenous women [49]. These associations are likely to be due to both underscreening and a higher prevalence of risk factors such as smoking and high parity [50], and emphasise the need to achieve equitable vaccination coverage across all geographical areas and population groups.

Schools with low attendance rates had similar odds ratios and school-level attributable risk percentage to schools with higher Indigenous enrolments. Two other Australian studies in recent years have also shown absenteeism to influence HPV vaccination coverage rates, specifically HPV vaccination completion coverage [30, 51]. High absenteeism rates reduce collective opportunities to initiate and complete the HPV vaccination course in school, and novel means of reaching and vaccinating adolescents with lower school attendance in other settings they engage with may be needed.

Special education schools were 5.6 times more likely to have low initiation coverage, with a median vaccination initiation coverage of only 53%; however, these schools only accounted for 8% of all schools in the three states, and thus had a school-level attributable risk of 27%. A recent review of vaccination in people with disability found 14 of 18 included studies reported that people with disabilities have lower rates of vaccination uptake across a range of different vaccines [52]. There have been only two Australian studies that have examined immunisation uptake in adolescents: one study on HPV vaccination [53], and one study on dTpa and HPV vaccination [54]. Both studies indicated that many young people with disabilities in Australia are missing out on adolescent vaccinations, with cited barriers including absenteeism, lack of consent and inability to immunise due to behaviour challenges [53, 54].

In this study, we calculated the school-level attributable risk to determine the school characteristics that contributed most to lower HPV vaccination initiation coverage. These outputs are consistent with a utilitarian ethical framework, where decisions are based on the greatest amount of benefit expected for the greatest number of individuals [55], in this case to reduce school-level variation in vaccination coverage and aim for the global target of 90% HPV vaccination coverage [56]. An alternative approach that could be utilised is the deontological ethical framework, which in this analysis would focus on the odds ratio only. A deontological ethical framework views ethics as a duty where the morality of an action, in this case providing vaccination to those in marginalised groups (even if it is a smaller group), is an obligation, and to not do so would be unacceptable, even if that meant limiting resources more broadly [55]. For example, this would mean we may focus on adolescents with disabilities in special education schools or Indigenous adolescents due to their well-known social and health disadvantages and the higher odds ratios. For Indigenous adolescents, addressing these variations in HPV vaccination uptake is important due to the disparities in the burden of disease from cervical cancer in Indigenous women [57]. For adolescents with disabilities, despite evidence that their sexual health needs are often similar to or greater than those of their typically developing peers, access to and utilisation of other forms of prevention including cervical screening is less common [52, 58]. A combination of the utilitarian and deontological ethical frameworks would be ideal to maximise coverage broadly and ensure equity for all populations.

Our study has a few limitations to consider when interpreting the findings. First, we used an ecological approach to identify schools and specific characteristics associated with lower HPV initiation; therefore, our approach does not assess risk factors at the individual level, but rather provides an indication of school-level factors that can inform interventions and future research. The covariates were based on those routinely available in a range of datasets, and some may have been markers of other risk factors. Many of the variables describing school characteristics were highly correlated, which may have led to an inability to resolve confounding and distinguish causally related factors. Second, due to the strong collinearity/multicollinearity between the school-level characteristics, we were unable to develop multivariable models without introducing bias of unpredictable direction and magnitude due to multicollinearity. Third, we were unable to include all special education schools, with the limited inclusion of ungraded special schools in our study; therefore, the estimated odds ratios may be much higher or lower than reported here. Finally, our analysis focused on the ability of the school-based vaccination programs to vaccinate students in the school setting and therefore did not include vaccinations that may have been administered later through GP clinics, which means HPV vaccine initiation coverage may be higher than reported in our study. Overall, HPV vaccination coverage in Australia is high and coverage trends from 2017–2020 were consistent with the findings of the study with coverage relatively resilient in the face of the COVID pandemic [16]. As our study results were based on data from 2016/2017, there may have since been some fluctuations in the prevalence of risk factors and how they contribute to lower HPV vaccination initiation coverage at the school-level. Additionally, as only three states in Australia participated in the study, the results may not be generalizable to all Australian states and territories although there are strong similarities in the way that schools operate and in how the vaccination program is delivered in schools across the country. Many of the factors identified as associated with lower school level coverage here have been noted previously to be individual student or school level risk factors in other jurisdictions of Australia e.g. students in special schools in Victoria [51] and school absenteeism in South Australia.

In conclusion, we determined that smaller schools, schools with a higher proportion of Indigenous enrolments and schools with lower attendance rates had lower odds than special education schools of having low HPV vaccine initiation coverage. These schools made up 33–79% of schools overall with lower vaccination initiation coverage, thus focusing initiatives in schools with these characteristics would reduce the greatest amount of school-level variation in these three jurisdictions. There is also a need to ensure equitable access to adolescent vaccination across low coverage schools, which, based on the odds ratio, would mean focusing on special education schools. Although school-based HPV vaccination programs have been successful in achieving high rates of vaccination coverage in adolescents in many countries, each country varies in its approach to delivery and prioritisation [59, 60], and coverage achieved is highly variable. The findings of this study may be particularly beneficial for countries with the lowest HPV vaccination uptake. Tailored strategies are critical to improving vaccination uptake in under immunized groups. A recent study in the UK found that new consent procedures can improve uptake of HPV vaccination [59]. Other studies have utilized enhanced reminders, educational and communication activities, and multicomponent strategies [60]. Other interventions that have been shown to improve HPV vaccination coverage included financial incentives and reminders, motivational behavioural interventions, training including assessment and feedback, and consultations [61]. Strategies need to be adaptable to specific contexts to maximize vaccination uptake and understanding school factors can help guide the application of these strategies. The findings from this study may help inform immunisation program planning and prioritisation of resource allocation to support school-based vaccination programs. Further qualitative research is also needed in these settings to understand barriers to vaccination and guide specific strategies to improve uptake in schools with these characteristics.

## Supplementary Information

**Additional file 1.** Sample SAS code for calculating the population attributable risk, using school size as example variable.  

## Data Availability

The data that support the findings of this study are available from the four statutory bodies [participating jurisdictional departments, ABS, ACARA and NHPVR] but restrictions apply to the availability of these data, which were used under license for the current study, and so are not publicly available. Data are however available from the authors upon reasonable request and with permission from the statutory bodies.
