# Supplementary material for: Measuring school level attributable risk to support school-based HPV vaccination programs
Source: BMC Public Health. 2022 Apr 25;22:822. doi: 10.1186/s12889-022-13088-x (PMC9036743; doi:10.1186/s12889-022-13088-x)
Supplement: Supplementary file 1 — Additional file 1. Sample SAS code for calculating the population attributable risk, using school size as example variable. [file 12889_2022_13088_MOESM1_ESM.docx]

**Appendix 1. Sample SAS code for calculating the population attributable risk, using school size as example variable**

Program:

title 'make variance-covariance matrix of beta coefficients';

**PROC** **LOGISTIC** DATA=TEMP1 COVOUT OUTEST=DATA.BETA;

MODEL OUTCOME=

school_size_small /* yes/no */

school_size_medium /* yes/no */

school_size_large /* reference */

title 'make dataset of joint prevalences of risk

factors';

**PROC** **SORT** DATA=all; By

school_size_small

school_size_medium

;

run;

**PROC** **MEANS** NOPRINT DATA=all ; VAR id;

OUTPUT OUT= PREV N=fq;

By school_size_small

school_size_medium ;

run;

**call the MACRO;

%***hwpar***(bdata= BETA,

pdata= PREV,

n_or_p=n,

n_or_pname=FQ,

MODVAR=

school_size_small /* yes */

,

FIXEDVAR=

school_size_medium /* no */

,

);

Output:

Full PAR (95% CI) for school_size_small school_size_medium

0.79 (0.757, 0.82 )

Partial PAR (95% CI) for

modifiable vbls :

**school_size_small**

fixed vbls :

0.564 (0.526, 0.602 )

Partial PAR (95% CI) for

modifiable vbls :

**school_size_medium**

fixed vbls :

0.226 (0.2, 0.255 )
